# SARS-CoV-2 Cellular Infection and Therapeutic Opportunities: Lessons Learned from Ebola Virus

**DOI:** 10.3390/membranes11010064

**Published:** 2021-01-18

**Authors:** Jordana Muñoz-Basagoiti, Daniel Perez-Zsolt, Jorge Carrillo, Julià Blanco, Bonaventura Clotet, Nuria Izquierdo-Useros

**Affiliations:** 1 IrsiCaixa AIDS Research Institute, Germans Trias I Pujol Research Institute (IGTP), Can Ruti Campus, 08916 Badalona, Spain; jmunoz@irsicaixa.es (J.M.-B.); dperez@irsicaixa.es (D.P.-Z.); jcarrillo@irsicaixa.es (J.C.); jblanco@irsicaixa.es (J.B.); BClotet@irsicaixa.es (B.C.); 2 Infectious Diseases and Immunity Department, Faculty of Medicine, University of Vic (UVic-UCC), 08500 Vic, Spain; 3 Infectious Diseases Department, Germans Trias i Pujol Hospital, 08916 Badalona, Spain

**Keywords:** SARS-CoV-2, Ebola virus, endocytosis, antivirals, antibodies, vaccines

## Abstract

Viruses rely on the cellular machinery to replicate and propagate within newly infected individuals. Thus, viral entry into the host cell sets up the stage for productive infection and disease progression. Different viruses exploit distinct cellular receptors for viral entry; however, numerous viral internalization mechanisms are shared by very diverse viral families. Such is the case of Ebola virus (EBOV), which belongs to the filoviridae family, and the recently emerged coronavirus SARS-CoV-2. These two highly pathogenic viruses can exploit very similar endocytic routes to productively infect target cells. This convergence has sped up the experimental assessment of clinical therapies against SARS-CoV-2 previously found to be effective for EBOV, and facilitated their expedited clinical testing. Here we review how the viral entry processes and subsequent replication and egress strategies of EBOV and SARS-CoV-2 can overlap, and how our previous knowledge on antivirals, antibodies, and vaccines against EBOV has boosted the search for effective countermeasures against the new coronavirus. As preparedness is key to contain forthcoming pandemics, lessons learned over the years by combating life-threatening viruses should help us to quickly deploy effective tools against novel emerging viruses.

## 1. Introduction

SARS-CoV-2 was identified on 7 January, 2020 as the etiological agent responsible for COVID-19, a severe respiratory disease currently causing a global pandemic. Since then, research groups worldwide have dedicated their efforts to understand the viral cycle of this new coronavirus and to find strategies to prevent infection. As of 12 December 2020, there were almost 70 million cases confirmed and more than 1.5 million deaths affecting 220 countries in the globe, sparkling global concern (https://www.who.int/emergencies/diseases/novel-coronavirus-2019?gclid=Cj0KCQiA8dH-BRD_ARIsAC24umbD-JsU2gwShKk7Q6H1RJ-lo0JZuRG8to08SFLhF6BL1YuRf4I-lHYaAn9aEALw_wcB). The high transmissibility of the virus, the broad range of symptoms associated to the disease and the lack of effective therapeutics to prevent the course of the infection has sped up the search for novel treatments and vaccines.

A similar challenge was faced by the scientific community between 2013 and 2020, when Ebola virus (EBOV) threatened humankind causing two major outbreaks in Central and West Africa, which caused an Ebola Virus Disease (EVD) that presented up to a 90% case-fatality rate. The incredible amount of scientific knowledge generated during the EBOV epidemic identified antivirals displaying efficacy against different steps in the EBOV life cycle, therapeutic neutralizing antibodies, and vaccine strategies. All these tools laid the foundations to better cope with future viral zoonotic infections. Some of these strategies have also been deployed against SARS-CoV-2, and the early efficacy shown in vitro has demonstrated key similitudes between both zoonotic viruses. In this review, we compare the life cycle of the filovirus EBOV and the beta-coronavirus SARS-CoV-2, which are very distant RNA-based viruses, focusing on the therapeutic strategies that tackle key steps shared by both viruses. Similitudes between EBOV and SARS-COV-2 highlight the importance of applying previous knowledge and key tools generated in preceding epidemics as a way to boost preparedness and confront new emerging viruses.

## 2. Setting the Stage for Infection: Viral Binding and Host Attachment Receptors for EBOV and SARS-CoV-2

**Figure 1:**
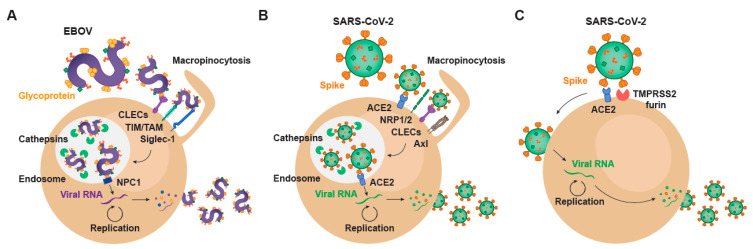
Viral and host factors involved in EBOV and SARS-CoV-2 infectious cycle. (**A**) EBOV entry into target cells is mediated by macropinocytosis, which directs surface-attached viral particles to the endosomal trafficking pathway. Within endosomes, host cathepsins cleave viral glycoprotein, facilitating interaction with the NPC1 receptor and viral membrane fusion. In the cytoplasm, the viral RNA genome undergoes transcription/replication, resulting in the synthesis of new viral particles that exit infected cells through membrane budding. (**B**) SARS-CoV-2 can enter target cells through an endosomal pathway that parallels EBOV internalization. Within endosomal compartments, cleavage of the Spike protein results in viral fusion and cytoplasmic entry, where viral replication occurs. (**C**) SARS-CoV-2 also enters target cells through an alternative mechanism in which Spike protein is cleaved at the cell surface, a process mediated by proteases such as TMPRSS2 and furin. In this case, the viral genome gains access to the cytoplasm through viral fusion with the plasma membrane. EBOV: Ebola virus; CLECs: C-type lectin receptors; TIM: T-cell immunoglobulin and mucin receptors; TAM: Tyro3-Axl-Mer receptors; Siglec-1: sialic acid-binding Ig-like lectin 1; NPC1: Niemann-Pick receptor C1; SARS-CoV-2: severe acute respiratory syndrome coronavirus 2; ACE2: angiotensin-converting enzyme 2; NRP1/2: neuropilin 1/2; TMPRSS2: transmembrane protease serine 2.

The very first step of the viral life cycle is the attachment of the virus via key receptors, followed by a viral entry process that relies on the same or alternative host factors that finally lead to productive infection. The availability of these critical host attachment molecules determines the tissue tropism, which greatly varies depending on the type of virus. Since the specific steps of viral binding and subsequent entry are shared among very distant viruses, lessons learned in the past can illuminate how a new virus like SARS-CoV-2 interacts with target cells. The spectrum of cellular molecules that act as virus attachment receptors is extremely broad, and viruses mostly can bind to more than one factor on the host cell membrane (Figure 1). Such is the case of EBOV, whose affinity to a wide variety of host cell receptors mediates viral binding to different cellular targets (Figure 1A). C-type lectins (CLECs), which are able to interact with particular glycans exposed on the viral glycoproteins, comprise DC-SIGN (dendritic cell-specific intercellular adhesion molecule-3-grabbing non-integrin), L-SIGN (liver/lymph node-specific intercellular adhesion molecule-3-grabbing non-integrin), hMGL (human macrophage galactose- and N-acetylgalactosamine-specific C-type lectin), and mannose-binding lectins, all of which bind to *N*- and *O*-linked glycans on Ebola virus glycoprotein, as reviewed in [1]. However, cells lacking CLEC expression remain permissive for EBOV infection. Importantly, phosphatidyl serine (PtdSer) binding receptors can also recognize this lipid exposed on the viral envelope of EBOV. PtdSer-recognizing receptors include protein complexes composed of Gas6 or protein S, members of the T-cell immunoglobulin and mucin domain (TIM) family TIM-1 and TIM-4, and the TAM family of receptor tyrosine kinases Tyro3, Axl, and Mer [2] (Figure 1A). EBOV binding efficiency also depends on the presence of plasma membrane sphingomyelin, and the activity of acid sphingomyelinase (ASMase) [3]. In activated myeloid cells, EBOV entry is enhanced by the sialic acid-binding Ig-like lectin 1 (Siglec-1/CD169), which recognizes sialylated gangliosides exposed on the cellular-derived membrane of the virus [4] (Figure 1A). Overall, these cellular receptors contribute to EBOV attachment and promote subsequent infection.

On the contrary, SARS-CoV-2 attachment to susceptible cells remains primarily on the binding of the Spike protein to the host angiotensin converting enzyme 2 (ACE2) on the susceptible cell membrane [5] (Figure 1B,C), and most importantly, ACE2 is also critical for viral fusion [6]. However, other factors may also actively interact with SARS-CoV-2 and promote viral binding and attachment. Clausen and colleagues demonstrated that heparan sulfate, which is a highly negatively charged polysaccharide attached to proteoglycans found on the cellular membrane or the extracellular matrix, interacts with the ectodomain of the SARS-CoV-2 Spike protein to shift its conformation and allow binding to ACE2 [7]. Integrins are also proposed as potential players in the entry of SARS-CoV-2 into the host cell [8], and the Spike protein has a specific motif able to bind these receptors. Integrin alfa and beta molecules recognize specific motifs in the spike protein of SARS-CoV-2 and have the potential to trigger infection by binding integrin heterodimers, activating transducing pathways involving phosphatidylinositol-3 kinase (PI-3K) or mitogen-activated protein kinase (MAPK), which can promote viral entry [8]. Thus, as already reported for EBOV, binding to integrins can facilitate SARS-CoV-2 endocytosis and infection. Neuropilin-1 and 2 (NRP1 and NRP2) have also recently been reported to play a role on SARS-CoV-2 attachment [9,10] (Figure 1B). Although the absence of these proteins still allows for viral entry into susceptible cells, infectivity gets reduced. Daly et al. hypothesize that the upregulation of neuropilins in lung tissues of COVID-19 patients and their binding to the SARS-CoV-2 spike protein may be one of the reasons to explain why this virus is more infectious than SARS-CoV-1 [10]. Another potential receptor for SARS-CoV-2 is the CD147 or extracellular matrix metalloproteinase inducer (EMMPRIN), a protein that belongs to the immunoglobulin superfamily enrolled in inflammatory processes and viral cellular entry [11].

Many of the early events that govern attachment of SARS-CoV-2 to cellular targets remain still unknown and require further investigation. CLECs already implicated in EBOV binding, such as DC-SIGN and L-SIGN, have also been associated with the capacity to transmit SARS-CoV-2 pseudoviruses to target cells expressing ACE2 [12] (Figure 1A,B). Moreover, SARS-CoV-2 specifically interacts with tyrosine-protein kinase receptor UFO (Axl) on the host membrane, where this receptor can promote viral entry [13], as already described for EBOV (Figure 1A,B). Interestingly and also previously reported for EBOV, fluoxetine, a functional inhibitor of ASMase, efficiently abrogates the SARS-CoV-2 entry and propagation in Vero E6 and CaLu-3 cells, suggesting that ASMase may also play a significant role in the early steps of the virus infection cycle [3,14]. A further understanding of the role of attachment factors implicated in SARS-CoV-2 binding will be required to reduce systemic dissemination between susceptible cells and tissues. Moreover, studying how these attachment factors set up the stage and facilitate viral entry and fusion will be critical to develop effective antiviral strategies.

## 3. EBOV Entry Converges with the Endosomal Route of SARS-CoV-2

Distinct viruses have evolved to use endocytic pathways to promote efficient infection, which requires the delivery of the viral genome into the cell cytoplasm at sites where replication proceeds optimally. Both SARS-CoV-2 and EBOV can utilize analogous pH-dependent endocytic routes to enter the cytoplasm of infected cells, since their viral proteins rely on similar proteolytic cleavage mechanisms that can take place at endosomal compartments. In the particular case of SARS-CoV-2 though, alternative entry processes at the plasma membrane are also key determinants of the pathogenesis of this coronavirus, as we will later discuss.

SARS-CoV-2 viral entry is mediated by the interaction of the Spike viral protein with ACE2, that allows for viral fusion and infection [6]. The Spike protein is comprised of two major units. The N-terminal S_1_ subunit contains the receptor binding domain (RBD), which is essential for attachment to ACE2. The C-terminal S_2_ subunit harbors key domains that play a role in membrane fusion and intracellular trafficking into the cytoplasm [15]. As reported in previous coronavirus studies, the cleavage of the Spike protein at the boundary between the S_1_ and S_2_ subunits by cellular host proteases is required for the activation of the protein to promote virus–cell fusion [16,17]. Indeed, there is an additional furin-type cleavage site at the junction between S_1_ and S_2_ of the newly discovered coronavirus that was not originally present in SARS-CoV-1, and is assumed to comparatively enhance SARS-CoV-2 infectivity [9,18]. Following the cleavage by furin of the S protein, the RBD of the S_1_ subunit of SARS-CoV-2 binds to the outer surface of ACE2 with a higher affinity compared to SARS-CoV-1 RBD [19,20]. This engagement triggers a conformational rearrangement that causes S_1_ shedding, cleavage of the S_2_ subunit by host proteases and exposure of a fusion peptide located next to the proteolytic side in S_2_ [17,21,22].

While the novel coronavirus mainly fuses at the cellular membrane of susceptible cells, where particular host proteases with the capacity to prime the Spike protein such as TMPRSS2 or TMPRSS4 are exposed, this virus can also exploit an alternative endocytic route [23] (Figure 1B). In certain cellular types, SARS-CoV-2 can also enter the cells via intracellular endosomal compartments, where other host proteases such as cathepsins can prime the Spike and promote viral fusion with internal endosome membranes [6]. This later endocytic route clearly resembles to that followed by EBOV, which is also internalized through an endosomal pathway that triggers viral fusion (Figure 1A,B). Following virus–cell attachment, EBOV is internalized primarily by macropinocytosis [24] (Figure 1A). Although other routes of uptake have been reported, including caveolin- and clathrin-dependent endocytosis, many of those studies have been performed with retroviral pseudotypes, which in the case of EBOV, do not display native virus morphology nor viral glycoprotein density and other biochemical characteristics [25].

As it happens with the Spike protein of SARS-CoV-2, EBOV contains a viral glycoprotein at the outer surface that mediates virus and host membranes fusion upon cellular protease cleavage. The mature conformations of GP with capacity to fuse with endosomal membranes requires a post-translational furin cleavage. This process produces a disulfide heterodimer composed of GP_1_ and GP_2_ subunits, being the former required for receptor interactions and the latter required for membrane fusion [26]. After initial internalization, virus particles are trafficked to the late endosomes/lysosomes through the endo-lysosomal pathway, where pH decreases and cysteine proteases cathepsins B/L cleave EBOV GP_1_ into its fusogenic form, which has the RBD exposed [27,28,29] (Figure 1A). Cathepsins L and B where initially identified as the essential proteases for the processing of EBOV GP and, indeed, their cleavage sites within the viral glycoprotein sequence have been mapped. The processed GP_1_ interacts with the late endosomal/lysosomal Niemann-Pick C1 (NPC1) intracellular receptor, which triggers the fusion of the viral envelope with the cellular endosomal membrane upon GP_2_ dependency [30,31] (Figure 1A). Although the specific mechanism is still not clear, the membrane fusion step also requires the activity of the Two-Pore Calcium Channel 2 (TPC2) in the endosomal membrane [32].

In the case of SARS-CoV-2, this endosomal viral entry pathway requires the binding of the Spike protein to ACE2 and its priming by cathepsin proteases [33] (Figure 1B). Thus, the cathepsin-mediated cleavage is a critical step for the entry of SARS-CoV-2 and EBOV. It is important to remember, however, that in contrast to EBOV, which can only fuse in endocytic compartments, SARS-CoV-2 mainly exploits the plasma membrane for accessing cellular targets in which specific serine proteases are able to prime the Spike of the coronavirus at the plasma membrane (Figure 1C). Proteolytic cleavage of the Spike protein by TMPRSS2 allows fusion at the plasma membrane of key cellular targets. As we will later discuss, this complicates the clinical use of cathepsin inhibitors and therapeutic agents that interfere with the endocytic route of entry for SARS-CoV-2, which displays an independent viral fusion pathway at the plasma membrane that is highly active in pulmonary cells [6]. Studying cellular gateways exploited by very distant viruses may aid to identify hot spots where viral entry converges, what will be key to develop broad pan-antiviral strategies aimed at avoiding infection. Once viral fusion takes place, productive infection will trigger viral replication and complicate viral control.

## 4. Transcription and Replication of EBOV and SARS-CoV-2

Once EBOV and SARS-CoV-2 genomes are released into the cell cytoplasm, viral replication occurs through a tightly regulated process involving viral and host factors (Figure 1). As both EBOV and SARS-CoV-2 are single-stranded RNA viruses, they share common features in their transcription and replication processes. However, the opposite polarity of their genomes also implicates the existence of relevant divergences between them. In this section, we will analyze the differences and similarities for EBOV and SARS-CoV-2 transcription and replication.

EBOV negative-sense RNA genome enters the cytoplasm in the form of a ribonucleoprotein complex. Viral genome is encapsidated by EBOV nucleoprotein (NP), and it is associated to the RNA-dependent RNA polymerase (L) and viral proteins 35 (VP35), 30 (VP30), and 24 (VP24), which play critical roles in viral transcription and replication. VP24 mediates viral uncoating, making the genome accessible to the transcription machinery [34,35]. VP35 and VP30 serve as co-factors for the L polymerase, that generates positive-sense mRNAs encoding the viral proteins using the viral genome as a template [36]. Following this primary transcription process, secondary transcription cycles are mediated by the newly synthesized viral polymerase and co-factors, thus amplifying the production and accumulation of cytoplasmic viral proteins [36] (Figure 1A).

In contrast to EBOV, coronaviruses have a positive-sense RNA genome that is readily translated by the host machinery upon cytoplasmic entry (Figure 1B,C). Translation of SARS-CoV-2 open reading frame 1a (ORF1a) and 1ab (ORF1b) results in the synthesis of the polyproteins 1a (pp1a) an 1ab (pp1ab), respectively [37]. These polyproteins need further processing to give rise to functional non-structural proteins 1-16 (nsp1-16), which contribute to the formation of a replication complex observed in other coronavirus species [38,39,40]. Moreover, they facilitate the synthesis of viral proteins by inhibiting the translation of host proteins [41,42]. Among non-structural proteins, the major protease nsp5 (M^pro^) and the papain-like protease nsp3 (PL^pro^) are the mediators of pp1a and pp1ab cleavage, which makes them essential for viral replication and attractive antiviral targets. Although there are no M^pro^ and PL^pro^ homologues in the EBOV genome, proteases are key molecules for other viruses such as the hepatitis C virus (HCV) and the human immunodeficiency virus type 1 (HIV-1) [43]. That was the reason why it was initially thought that repurposing of HCV and HIV-1 protease inhibitors could help to treat SARS-CoV-2 infection, but unfortunately this strategy failed to provide solid therapeutic candidates [44].

EBOV and SARS-CoV-2 protein synthesis is accompanied by the replication of the viral genome (Figure 1). In EBOV infection, the L polymerase copies the negative-sense RNA generating positive-sense antigenomes, which in turn serve as templates for the synthesis of new negative-sense genomes [36]. Similarly, the RNA-dependent RNA polymerase nsp12 generates full-length negative-sense copies of SARS-CoV-2 RNA, that can be copied for generating the new positive-sense genomes [45]. Therefore, both EBOV and SARS-CoV-2 rely on the activity of their RNA-dependent RNA polymerases as central molecules for viral replication. Thus, polymerases have been also considered major targets in the development of novel antiviral therapies for both viruses.

In addition to viral proteins, host factors play a role in the transcription/replication of viral genomes. For EBOV, the DNA topoisomerase I and the RNA-binding protein Staufen 2 participate in the synthesis of viral RNAs [46,47]. NXF1 and DDX39 are RNA splicing and export factors that contribute to viral transcription and translation [48], while the protein phosphatases 1 (PP1) and 2A (PP2A) activate VP30 through dephosphorylation [49,50]. Intriguingly, the host retinoblastoma-binding protein 6 (RBBP6) and the double stranded RNA-binding protein 76 (DRBP76) are host restriction factors that inhibit PP2A and L protein activity, respectively [47,51,52], which suggests the therapeutic potential of inhibiting these viral proteins. Although there is still a lack of information regarding host factors governing SARS-CoV-2 replication, knowledge gathered in the study of other coronavirus species could provide clues on the factors involved in SARS-CoV-2 replication. For example, some coronaviruses modify the phosphorylation of the eukaryotic initiation factor 2 (eIF2) to take control over host translation [53], highlighting the therapeutic potential of inhibiting this and other translation factors. The eukaryotic elongation factor 1A2 (eEF1A2) is also a very interesting candidate that has offered a new antiviral approach, as we will later discuss. Future studies will identify novel factors involved in SARS-CoV-2 transcription/replication, thus increasing the opportunity for therapeutic interventions.

Both EBOV and SARS-CoV-2 replicate in particular cellular localizations. EBOV replicates in inclusion bodies whose formation relies on the presence of viral NP and host importin-α7 [54]. Similarly, coronavirus replication occurs in specialized compartments termed replication organelles formed in the presence of nsp3, nsp4, and nsp6 viral proteins [55,56,57]. The connection between EBOV-driven inclusion bodies and the replication organelles observed in SARS-CoV-2-infected cells remains uncertain. However, the later seem to play an important role in SARS-CoV-2 replication [58], so further research in this field is guaranteed. Taken together, a number of viral and host factors play key roles during the process of viral transcription and replication of SARS-CoV-2 (Figure 1), and some of them have homologous counterparts in the infection by EBOV and other viruses, such as the viral polymerase and proteases. Tackling these factors could prevent cytoplasmic accumulation of newly synthesized proteins and viral genomes, which leads to the assembly and egress of new virions.

## 5. EBOV Egress and Common Gateways to SARS-CoV-2

The final steps of the EBOV and SARS-CoV-2 infectious cycle consist in the assembly of viral proteins around the nucleocapsid and the budding of new virions from the plasma membrane (Figure 1). Again, both processes are regulated by a spectrum of viral and host factors, whose interaction will define the production of infectious particles derived from newly generated viral proteins and genomes that accumulate in the cytoplasm following replication. Newly generated EBOV genomes associate with NP in a process of ribonucleoprotein condensation that is mediated by VP24 [34,35]. Genomes also associate with L protein, VP35 and VP30 to ensure the initiation of the next replicative cycle, and then migrate to the plasma membrane through a mechanism involving the actin cytoskeleton [59,60]. The assembly of the virion is mediated by VP40 [61], which interacts with different trafficking components to reach the membrane, including actin filaments [62,63], microtubules [64], and the COPII vesicle system [65]. In addition to the ribonucleoprotein complex and VP40, GP is transported to the assembly sites through the endoplasmic reticulum (ER)–Golgi secretory pathway. During trafficking, GP undergoes post-translational modifications including *O-* and *N-*glycosylation and the furin-mediated cleavage of subunits GP_1_-GP_2_ [26], which is dispensable for viral infectivity [66,67].

The host factors that participate in EBOV assembly and budding include a number of components of the endosomal complex required for transport (ESCRT), such as the tumor susceptibility gene 101 (TSG101) and the vacuolar protein sorting-associated 4 (VPS-4) [68]. In addition, the ubiquitin ligase Nedd4 participates in viral egress through interaction with the ESCRT machinery [68]. Viral budding occurs in cholesterol-enriched domains of the plasma membrane containing gangliosides [69], which allows viral interaction with ganglioside-binding receptors such as Siglec-1 [4]. Host scramblases also modify the viral membrane lipidomics, exposing phosphatidylserine on the viral surface [70], thus enabling interaction with phosphatidylserine receptors as well [71].

The capacity of SARS-CoV-2 to interact with such receptors remains unclear, as many of the aspects involving viral assembly and egress. SARS-CoV-2 contains the structural envelope (E), membrane (M), and nucleocapsid (N) proteins, as well as the Spike glycoprotein. In other coronaviruses, N encapsidates the genome, while M and E ensure that the nucleocapsid is incorporated to the nascent virion. However, the particular implications of these proteins during SARS-CoV-2 assembly needs further investigation. Viral budding of other coronaviruses occurs through the ER-Golgi exocytosis pathway [72,73], and preliminary data suggests that this might also be the case for SARS-CoV-2 [74]. The host factor CD74 can block the activity of cathepsins and is expressed in the ER membranes of immune cells, where it facilitates the export of MHC-II from the ER towards vesicles that fuse with the late endosome. In particular, the inhibition of cathepsins is mediated by the thyroglobulin domain of CD74 [75], which can abrogate the cleavage and processing of different viral glycoproteins and has an antiviral role in both EBOV and SARS-CoV-2 [76]. However, alternative data also points out that SARS-CoV-2 could employ the lysosomal trafficking pathway to exit infected cells [77]. Future research will be required to delineate the molecular mechanisms and the precise viral and host factors that mediate SARS-CoV-2 assembly and budding. Searching for common exit pathways shared by EBOV and SARS-CoV-2 could aid in the identification of novel antiviral strategies.

## 6. Repurposing Drugs against EBOV and SARS-CoV-2: A Shared Strategy to Find Effective Antivirals

SARS-CoV-2 set an unprecedented situation for the scientific community. A global pandemic caused by an infectious agent with a tremendous dissemination speed required urgent measures and therapeutics to counteract its impact on global health and economy. In the race to find a cure, the same strategy used against EVD during the 2013–2016 West African EBOV outbreak was used against COVID-19: antiviral screenings of repurposed drugs. The efficacy in vitro of some FDA-approved drugs allowed the identification of viral replication mechanisms of EBOV and the selection of specific inhibitors, setting up a faster and cheaper strategy for the study of emergent viruses such as SARS-CoV-2.

**Figure 2:**
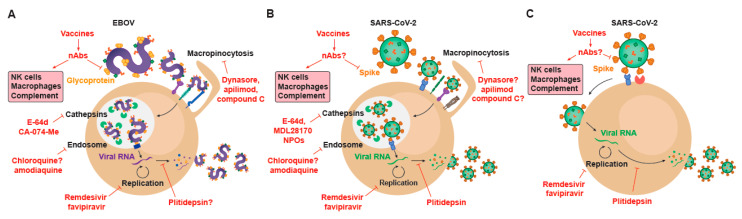
Antiviral strategies aimed at blocking EBOV and SARS-CoV-2 viral cycle. (**A**) Different antivirals tackle viral and host factors involved in EBOV replicative cycle. (**B**) Antivirals directed against the SARS-CoV-2 endocytic pathway. Several of these antivirals have also been proposed to block EBOV entry through similar mechanisms. (**C**) Antiviral strategies targeting SARS-CoV-2 cycle in cells undergoing viral fusion at the plasma membrane. nAbs: neutralizing antibodies. Immunological antiviral functions of neutralizing antibodies (NK- or complement-mediated infected cell lysis and macrophage phagocytosis) are highlighted in boxes.

Viral factors implicated in cellular entry have provided several antiviral targets in vitro (Figure 2), although their role on relevant cellular functions has also complicated their downstream clinical application. Indeed, several inhibitors of macropinocytic processes have reduced filoviral entry in vitro. These include dynasore, which blocks dynamin; apilimod; which inhibits PIKfyve (phosphoinositide kinase, FYVE-type zinc finger containing), and the AMPK (AMP—activated protein kinase) inhibitor, compound C (Figure 2A). Interestingly, in one of the broadest studies of antivirals against SARS-CoV-2 published so far, apilimod was also identified as a promising agent that inhibited the entry of the new coronavirus into Vero E6 and also abrogated viral replication in a primary human lung explant model [78] (Figure 2B). Aplimod has been tested in patients with Crohn’s disease to decrease IL12/23 secretion, and has showed mixed clinical outcomes in phase I/II and phase II trials [79]. Yet, in all these clinical trials, it was generally well-tolerated [79]. However, to the best of our knowledge, this antiviral has not demonstrated efficacy yet neither in animal models nor in COVID-19 related clinical trials, although it has shown efficacy at abrogating viral entry for both EBOV and SARS-COV-2 in a side to side cellular comparison in vitro [80].

In addition, antimalarials that accumulate in endolysosomes, such as chloroquine, have protected mice against EBOV challenge [81], although in subsequent studies it failed to protect guinea pigs, mice, and hamsters [82,83,84] (Figure 2A). Artesunate-amodiaquine is another antimalarial that was associated to protection against EBOV during the 2013–2016 West African outbreak [85] (Figure 2A). Since the early onset of the SARS-CoV-2 pandemic, hydroxychloroquine was very rapidly suggested as a potential candidate to combat the infection of the new coronavirus. Initially it was proposed that chloroquine could directly interfere with clathrin-mediated endocytosis of SARS-CoV-2 [86] or even block SARS-CoV-2 spike interaction with GM1 gangliosides on the plasma membrane [87], as gangliosides were previously shown to bind to SARS-CoV-1 Spike protein [88]. However, it was later demonstrated that hydroxychloroquine exerts its activity against SARS-CoV-2 by disrupting the viral entry via the endocytic endosomal pathway in Vero E6 [89,90] (Figure 2B). Once internalized within early endosomes, cathepsins cleave the glycoprotein of EBOV, allowing for viral fusion through the interaction with the NPC1 receptor and the release of the viral genome into the cytoplasm (Figure 1A). Importantly, several cathepsin inhibitors, including E-64d and CA-074-Me, inhibit EBOV entry into target cells [91] (Figure 2A). As we have already discussed, a similar entry mechanism has been reported for SARS-CoV-2, where viral trapping in endosomes allows for the release of the coronavirus RNA into the cytoplasm upon cathepsin cleavage of the Spike protein. Indeed, several cathepsin inhibitors also block SARS-CoV-2 cellular entry via the endosomal route in Vero E6, including E-64d [6] but also other compounds such as MDL28170 [78] or NPO agents [92] (Figure 2B). Although these cathepsin inhibitors are all in pre-clinical stage, they have clearly shown potent activity abrogating viral entry in vitro in Vero E6 and HEK-293T cells transfected with ACE2. However, the most prominent entry mode of SARS-CoV-2 in pulmonary cells relies on viral fusion with the plasma membrane [6] (Figure 1C). This is possible due to the activity of cellular proteases such as TMPRSS2 and other members of this protease family such as TMPRSS4 [23], that act on the plasma membrane after viral binding to ACE2 receptor to prime the Spike protein of the coronavirus and trigger viral fusion (Figure 1C).

In cells expressing TMPRSS2, SARS-CoV-2 fusion with the plasma membrane renders cells insensitive to the action of compounds that interfere with the endosomal entry pathway. Thus, although hydroxychloroquine and cathepsin inhibitors block viral entry via the endosomal pathway in kidney cell lines such as Vero E6 or HEK-293T [92], this viral entry route is absent in pulmonary cells that are the primary targets of SARS-CoV-2 infection [93]. These findings can explain why in animal studies hydroxychloroquine has failed to protect infected macaques [93] and why in randomized clinical trials of hydroxychloroquine no significant protective effect has been observed in monotherapy [94,95,96]. Nonetheless, in combined therapies, it should be noted that agents targeting the alternative endosomal SARS-CoV-2 entry route such as hydroxychloroquine or MDL28170 could be key to stop viral dissemination in other extrapulmonary tissues where viral replication has already been detected [97], and viral entry could take place through this endosomal pathway. Overall, the different entry routes of SARS-CoV-2, depending on the cellular targets [98], indicate that complementary approaches will be needed to achieve a broad viral suppression in distinct tissues.

To date, remdesivir is the only approved antiviral drug for the specific treatment of COVID-19. The reason why this particular compound was quickly approved by the FDA and EMA is because remdesivir had previously shown clinical safety during the last EBOV outbreak [99]. Moreover, the mode of action of remdesivir had been clearly defined, acting as an adenosine analogue that is incorporated into nascent viral RNA chains and results in premature termination [100], a key feature that suggests the applicability of this antiviral as a broad-spectrum agent against different RNA viruses (Figure 2). When the SARS-CoV-2 pandemic began, it was already known that remdesivir was efficacious against other highly pathogenic coronaviruses such as SARS-CoV-1 and MERS-CoV [101], and that was the reason why it was quickly tested against SARS-CoV-2 in non-human primate animal models [102]. These promising results along with the clinical safety demonstrated during trials performed for EBOV prompted the WHO to include remdesivir in one of the four arms of the SOLIDARITY trial, in which hydroxychloroquine, ritonavir/lopinavir, and ritonavir/lopinavir plus ß-interferon regimes where also tested [94,103].

Unfortunately, although remdesivir has proven effective in randomized controlled trials for SARS-CoV-2 infected individuals [104,105], a recent update of the WHO open-labeled clinical trial has failed to detect any effect on the overall mortality in patients treated with remdesivir, and no changes in the initiation of ventilation and duration of hospital stays [94]. Results in clinical trials have therefore shown a limited therapeutic effect against the new coronavirus, a situation that resembles to that obtained during the EBOV outbreak of the Democratic Republic of Congo in 2019, where remdesivir showed a reduced effectivity at reducing mortality as compared to the groups receiving the antibodies against the glycoprotein of the Zaire strain MAb114 and REGN-EB3 [99]. Intriguingly, even in those studies where remdesivir has shown clinical benefit in COVID-19 patients, and also in the extremely well-controlled experimental infections performed in rhesus macaques, remdesivir has failed to demonstrate a significant reduction in viral load, which has always been the goal standard for any antiviral treatment that has proved efficacy. A clearer reduction in viral load in SARS-CoV-2 with an antiviral has been recently shown in the hamster animal model, but only when animals were treated with the nucleoside analogue favipiravir at very high doses [106]. Importantly, favipiravir had previously shown protection against EBOV in non-human primates [107] (Figure 2A). While several clinical trials studying favipiravir for COVID-19 are currently registered at clinicaltrials.org, to the best of our knowledge no final results analyzing large cohorts have been reported for this compound. Interference with the host factor eEF1A2 targeted by plitidepsin [108] has showed in vitro anti-SARS-CoV-2 activity [92] (Figure 2B,C), and is currently being tested in a phase I clinical trial (NCT04382066/APLICOV).

Beyond the use of classical antiviral compounds, clinically approved antibodies have also shown a tremendous potential to combat infectious agents, and are therefore a new strategy that is gaining momentum in the fight against the novel coronavirus. This strategy has been clearly boosted by results of recent clinical trials performed with specific antibodies targeting EBOV, which have demonstrated the utility of this strategy to treat infections caused by emergent viruses.

## 7. How the Development of EBOV Treatments Based on Monoclonal Antibodies Targeting the Virus Guides the Design of Novel Therapies for SARS-CoV-2

The classical immune antiviral defense is mediated by the innate Type-1 interferon responses and the adaptive second-line cellular immunity. However, humoral responses also play a relevant role and contribute to virus clearance. Certainly, the humoral branch of the immune system is able to elicit antibodies targeting the most abundant viral proteins only a few days after viral infection. In particular, 9 and 16 days after symptoms onset are required to reach 50% and full seroconversion after SARS-CoV-2 primoinfection, respectively [109].

Antibodies play a wide range of functions mediated by the variable regions that specifically bind to the antigen, and by the fragment crystallizable (Fc) moiety that interact with Fc receptors expressed on the surface of different immune cells to deploy effector antibody functions [110,111]. Given this dual activity, the most relevant antibodies are those recognizing outer viral glycoproteins, the GP and Spike proteins for EBOV and SARS-CoV-2 viruses, respectively [112,113], since they can directly target both viral particles and infected cells and activate the immune system. A subset of these antibodies recognizing key viral glycoprotein domains that mediate receptor binding or fusion steps may interfere with viral entry mechanisms and inhibit viral infection. These antibodies, called ‘neutralizing antibodies’, show antiviral activity [112,114], not only by acting as entry inhibitors, but also by displaying effector functions, thus becoming multifunctional antiviral agents (Figure 2). Particularly, IgG1 and IgG3 neutralizing antibodies activate complement-mediated lysis, antibody-dependent cellular cytotoxicity (ADCC) and antibody-dependent phagocytosis (ADCP), contributing to antiviral functions (Figure 2). Importantly, in several viral infections, immunocomplexes have been shown to enhance antigen uptake and presentation by antigen-presenting cells, allowing induction of stronger humoral and cellular antiviral immune responses, as reviewed in [115].

This plethora of functions make antibodies excellent drug candidates against different human diseases, including infectious diseases. Indeed, much before we gained knowledge on antibodies, serum therapy for diphtheria was already developed by Emil von Behring [116]. In addition, hyperimmune IgG preparations and treatment with convalescent plasma has been reported for a wide range of infectious diseases [117]. The rationale behind this strategy is that convalescent individuals have developed a protective humoral immune response containing neutralizing antibodies that may exhibit protective activity. However, it is not until recent development of monoclonal antibody isolation [118] and recombinant protein expression technologies [119], that antibodies have emerged as highly specific and potent drugs against several human diseases. Although most antibody-based drugs are successfully commercialized to treat hematological or solid tumors and chronic autoimmune diseases [120,121], infectious diseases are also emerging as a new field for monoclonal antibody therapy. For instance, these strategies are currently useful for the prevention of RSV infection (palivizumab) [122], for the development of new HIV eradication strategies [123] and for the treatment of EBOV disease [124].

In the particular case of EBOV disease, early works using non-human primate models showed the preventative and therapeutic capacity of both neutralizing antibody and plasma infusions [125]. These data led to the preclinical and clinical development of several monoclonal antibodies against GP [125]. The recent PALM clinical trial, a randomized controlled trial of EBOV disease therapeutics, has provided a definitive answer for the clinical efficacy of different candidates. The trial tested the triple monoclonal antibody ZMapp (as control), the antiviral agent remdesivir, the single monoclonal antibody MAb114, or the triple monoclonal antibody REGN-EB3. Both MAb114 and REGN-EB3 were superior to ZMapp in reducing mortality from EBOV disease [99]. Indeed, the REGN-EB3 became the first EBOV therapy approved by the FDA in October 2020 (https://www.fda.gov/news-events/press-announcements/fda-approves-first-treatment-ebola-virus). These differences in efficacy have been recently explained by a systematic analysis of monoclonal antibodies against EBOV GP, which was able to define the main features that contribute to protection, specifically neutralization via epitopes maintained on endosomally cleaved GP and effector functions mediated by antibodies closest to the GP apex [126].

Following the path of other human infections, a program for the development of antibody-based therapies has been opened by several companies and research centers to fight against SARS-CoV-2. In an unprecedented short period, neutralizing antibodies have been isolated and characterized, animal models have been explored to validate the therapeutic or preventative activity of antibodies, while convalescent plasma and recombinant monoclonal antibodies have reached clinical trials in humans, as reviewed in [127]. Early after the identification of SARS-CoV-2 as the etiologic agent of COVID-19, a large number of human monoclonal antibodies recognizing the RBD and other regions of the Spike glycoprotein were isolated through single cell B cell sorting [128,129]. The concomitant development of animal models of infection allowed for a rapid testing of their therapeutic potential. For example, a dose dependent protective activity was confirmed in Syrian Golden hamsters [130] and mice [131] when the antibodies were administered. All these data fostered clinical trials based on convalescent plasma therapy (as the fastest way to act) and clinical development of monoclonal antibodies. A relatively large amount of data is already available on convalescent plasma [132,133]; however, it is still unclear whether this approach offers a clinically significant benefit and whether this benefit could be more evident in critical or early treated patients. Similar questions are still open for monoclonal antibody therapy, which has been developed in parallel. Indeed, Lilly, Regeneron, and other pharma companies have started clinical trials with different neutralizing monoclonal antibodies either alone or in combination (as for EBOV disease) in COVID-19 patients. Current data suggest that antibodies are ineffective at demonstrating clinical efficacy in hospitalized patients, as demonstrated by the failure of bamlanivimab, the Lylli LY-CoV555 antibody clinical trial (https://www.the-scientist.com/news-opinion/eli-lilly-halts-antibody-trial-in-hospitalized-covid-19-patients-68090). Newly designed trials, and novel developed antibodies will be required to demonstrate efficacy of monoclonal antibodies in COVID-19 patients, as already shown for EBOV disease.

Monoclonal antibodies are effective drugs to prevent or treat infectious diseases and current technologies allow for their rapid development; however, we still need to improve screening criteria for selection of ideal candidates, to define the window of opportunity for their optimal clinical effect and to reduce production costs to fully exploit their potential in the field of infectious diseases. Moreover, since antibodies targeting EBOV GP [99] and Spike glycoproteins from SARS-CoV-2 [134] play a major role protecting from infection or disease, most vaccine designs against both viruses have focused on those viral proteins that are key to mediate viral entry into the host cells.

## 8. Vaccines for EBOV and for SARS-CoV-2 Infection

The EBOV outbreak that took place in 2013–2016 in some countries of West Africa (Guinea, Nigeria, Senegal, Mali, Sierra Leona, and Liberia) meant a turning point in clinical vaccine development. During that time, the clinical development of some EBOV vaccines was impressively sped up as never before. Indeed, there are five of them already licensed: GamEvac-Combi [135]-licensed for emergency use in the Russian Federation-; Ad5-ZEBOV [136]-licensed in China-; VSV-ZEBOV-licensed in Europe and USA from the end of 2019 with the commercial name of Ervebo-; Ad26.ZEBOV with MVA-BN-Filo-licensed in Europe with the name of Zabdeno and Mvabea, respectively, from the middle 2020. Moreover, other vaccines such as ChAd3-EBO-Z, that is co- administered with MVA-BN-Filo, are in clinical development [137]. All these vaccines have proved to be safe, immunogenic and protective from EBOV infection. However, they showed differences in immunogenicity, duration of immune response and correlates of protection, as reviewed in [138]. Studies performed in animal models pinpointed the importance of both humoral and cellular immune responses in protection against EBOV [139,140,141]. Furthermore, as already discussed, the passive infusion of neutralizing antibodies has shown efficacy in the treatment of EVD [99], reinforcing the importance of eliciting this sort of antibodies by vaccination (Figure 2). Accordingly, previous results obtained with EBOV survivors showed that these individuals developed a strong neutralizing humoral response to EBOV GP [142] that can be higher than the humoral response observed in deceased patients [143,144].

Currently, there is a race for the development of a SARS-CoV-2 vaccine. Less than one year from the discovery of SARS-CoV-2 as the causal agent responsible of an outbreak of pneumonia in Wuhan, China [145,146], about 201 vaccine projects are in development worldwide (WHO.DRAFT landscape of COVID-19 candidate vaccines–29 October 2020). Despite most of these projects being in preclinical stages (*n* = 156) or early clinical development (35 out of 201), a handful of them (*n* = 10) have reached Phase III clinical trials in a record time, as reviewed in [147,148]. Most of these highly advanced vaccines use the SARS-CoV-2 Spike as an immunogen, although three of them are based on whole inactivated viruses (Sinovac Biotech; Sinopharm/Wuhan Institute of Biological Products and Sinopharm/Beijing Institute of Biological products). From those using the Spike, several approaches have been followed, including non-replicative viral vectors, such as chimpanzee adenovirus (AstraZeneca/University of Oxford); adenovirus serotype 26 (Janssen Pharmaceutical); adenovirus serotype 5 (Cansino Biologics/Academy of Military Medical Sciences) or a combination of both (The Gamaleya National Research center for Epidemiology and Microbiology/Academy of Military Medical Sciences). Besides, two companies (Moderna and Pfizer/BioNtech) are using RNA delivery and Novavax is developing a vaccine based on nanoparticles adjuvanted with Matrix M [149]. Despite the lack of a clear correlate of protection, which has not been defined yet for SARS-CoV-2 infection, neutralizing antibodies have proved to be protective in animal models [129,134,150] and probably humans [151] for SARS-CoV-2 acquisition. Interestingly, all vaccines in Phase III have shown to be safe in humans and are able to induce a neutralizing humoral response. In most cases, the titer of these antibodies was similar or even higher than those observed in COVID-19 convalescent patients. Importantly, none of those vaccines has reported any case of vaccine-induced enhancement of the disease in vaccinated volunteers. 

The previous efforts to develop EBOV vaccines during the 2013–2016 outbreak have laid the foundations to produce novel vaccines against SARS-CoV-2 in the most expedited way ever accomplished by humankind. These vaccines will eventually lead to the control of the current SARS-CoV-2 pandemic in the near future, but will also reinforce our capacity to tackle forthcoming emergent pathogens.

## 9. Conclusions and Future Perspectives

EBOV set an unprecedented scenario in 2013 in West Africa, where humankind was globally threatened by a very infectious agent with up to a 90% case-fatality rate. The scientific community worldwide put efforts to speed up the research for effective therapeutics against EVD. Although there is an FDA-approved vaccine currently in use, there are only limited treatments in place. Similar challenges are faced nowadays against the new coronavirus.

Clinical vaccine development experimented a turning point with the EBOV African crisis that began in 2013. The already five licensed EBOV vaccines and the efficacy of infused neutralizing antibodies in patients with EVD shed light on effective strategies to follow in order to develop both humoral and cellular responses, and this knowledge has paved the way to test these strategies in COVID-19 patients as well in a record time. For SARS-CoV-2, there are at least 10 vaccines that have reached Phase III clinical trials, all of them showing promising results on safety and efficacy, and two of them using for the first time RNA delivery against an infectious pathogen. The experience gained during the last decade on vaccine vector platforms and the close collaboration between scientists and the regulatory authorities have been key for the rapid development of vaccine candidates that have very recently been approved.

The strategy followed against EVD during the EBOV West and Central Africa outbreaks was to repurpose broadly-acting antiviral compounds. The repurposed drugs have the advantage of (i) being already approved by the regulatory agents; (ii) having already known pharmacodynamic and pharmacokinetic properties; (iii) having established side effects; and (iv) having potential efficacy against a specific step in the viral life cycle. This strategy has also been deployed in the current SARS-CoV-2 pandemic, allowing the scientific community to identify and already approve for clinical use treatments such as remdesivir in less than six months. Future studies will most likely focus on the search and design of more specific antivirals, and indeed, specific neutralizing antibodies against SARS-CoV-2 are already being tested in the clinic. The example of an old viral enemy, such as EBOV, and a newly encountered threatening coronavirus beautifully illustrates why lessons learned studying viral infection are paramount to develop effective vaccines, antibodies and antivirals against a particular virus, but are also key elements to potentiate our preparedness to combat future pandemics.

## Data Availability

Not applicable.
